# Maize Yield and Planting Date Relationship: A Synthesis-Analysis for US High-Yielding Contest-Winner and Field Research Data

**DOI:** 10.3389/fpls.2017.02106

**Published:** 2017-12-21

**Authors:** Nguyen V. Long, Yared Assefa, Rai Schwalbert, Ignacio A. Ciampitti

**Affiliations:** ^1^Department of Agronomy, 2004 Throckmorton Plant Science Center, Kansas State University, Manhattan, KS, United States; ^2^Department of Food Crop Science, Vietnam National University of Agriculture, Hanoi, Vietnam

**Keywords:** maize, high yield, latitude, planting date, synthesis-analysis

## Abstract

For maize (*Zea mays* L.), early planting date could be of advantage to high yields but a review of planting date effect on high-yielding data is not yet available. Following this rationale, a synthesis-analysis was conducted from the farmer annual maize contest-winner data (*n* = 16171 data points; 2011–2016 period); cordially provided by the National Corn Growers Association and a scientific literature dataset collected from research publications since the last three decades. The main objectives of this study were to: (i) identify spatial yield variability within the high-yielding maize dataset; (ii) understand the impacts of planting date on yield variability; (iii) explore the effect of management practices on maize yield-planting date relationship, and (iv) utilize the yield-planting date dataset collected via farmer contest-winner as a benchmarking data to be compared to the compendium of scientific literature available for yield-planting date relationship for the primary US maize producing regions. Major findings of this study are: (i) significant correlation between planting date and latitude, (ii) maize yield was maximized when planting window was 89–106 day of the year (DOY) for the 30–35°N, 107–118 DOY for the 35–40°N, <119 DOY for 40–45°N, and <129 DOY for 45–50°N, and (iii) both, yield contest and literature datasets portrayed that planting date becomes a more relevant factor when planting late, presenting a relatively smaller planting window in high-compared to low-latitudes.

## Introduction

For maize (*Zea mays* L.), wise use of the planting date window, lengthening the growing season while exploring favorable conditions at critical crop growth stages, has been one of the main factors to be considered for high-yielding production. Earlier planting dates contributed to the increase of maize yield in different US growing regions (Cardwell, 1982; Bruns and Abbas, 2006; Kucharik, 2008). Increasing length of the growing season allows producers use high potential hybrids, apply N more efficiently, choose the right hybrid maturity to complete physiological maturity before killing frost (Kucharik, 2006), and optimize seeding rate or plant density, all management tools available to maximize capture of sunlight, biomass conversion, and consequently overall maize productivity. However, it is unclear if the planting date trend will keep progressing towards earlier dates in the future as this practice is also limited by lower soil temperatures and wet early season soil conditions (Kucharik, 2006, 2008; Cassman, 2016).

Relationship between planting date and yield, and the influence of other management factors including pest and diseases (Wiatrak and Wright, 2004; Bruns and Abbas, 2006), hybrid maturity (Lauer et al., 1999; Anapalli et al., 2005; Grassini et al., 2011), water availability (Norwood, 2001), tillage systems (Imholte and Carter, 1987; Perez-Bidegain et al., 2007), and plant density (Nafziger, 1994; van Roekel and Coulter, 2011; Assefa et al., 2016; Lindsey and Thomison, 2016) has been studied at certain locations with limited observation. Significant yield reductions were found when planting was delayed after late April in mid-South (Bruns and Abbas, 2006) and after June (instead of March) in southeast (Wiatrak and Wright, 2004) mainly due to more frequent influence of pest and diseases at late planting. In Corn Belt states, stronger adverse effect was found for delayed than advanced planting (Swanson and Wilhelm, 1996); longer maturity hybrids were more sensitive to late planting date than the short maturity hybrids. However, late planting (early May) produced greater yield than early planting (mid-April) in dryland conditions. Adopting suitable tillage systems was recommended when planting was early or late. Superior plant density and longer maturity hybrids could be used to increase yield when planting under favorable weather conditions for crop growth. Further attempts to understand planting date trends on multiple site-years (Kucharik, 2006, 2008; Abendroth et al., 2017) reported changes in the optimum planting window based on the region evaluated over the last decades. Selection of the optimum planting window will play a more critical role for future maize productivity, more specifically in the context of climate change (Abendroth et al., 2017; Seifert et al., 2017). Therefore, regional efforts will be required to better understand how the optimum planting window is changing across US maize growing latitudes and, at the same time, to identify solution addressing yield reduction if planting is forced to be too early or too late.

A recently published historical maize yield gain study for North America documented that, primarily, high and very high-yielding environments contributed to the yield progress during the last three decades (Assefa et al., 2017). The same authors found that US maize yield growth rate continues to increase up to present time (increase from 40 to 50% for 1987–1996 and from 75 to 80% for 2007–2015). Nonetheless, the main production factors contributing to yield gain were not explored in the abovementioned synthesis analysis. Thus, exploring the effect of management practices on maize yield and planting date relationship from farmer-driven yield contest data could provide an insight on the main management factors contributing to yield progress. Unfortunately, insufficient effort has been paid on the evaluation of farmer maize data available from yearly yield contest-winner national program yet.

To meet those aims, a high-yielding dataset was collected from the National Corn Growers Association (NCGA) yield contest-winners, covering 46 US states from 2011 to 2016 period. Yield contest data was reported to be two-to-three times greater than the statewide-yield (Duvick and Cassman, 1999) and could be comparable to potential yield - maximum yield reached by a crop in a given environment (Egli and Hatfield, 2014). The high-yielding contest-winner data can provide a new insight on partial maize yield variability and the underlying factors that ultimately provides opportunity for advancing future crop management in different US maize growing areas. In addition, a synthesis-analysis using yield and planting date dataset gathered from the published scientific literature for the last 3 decades (1979–2014) was performed to validate our findings from the farmer-driven high-yielding database. Following this rationale, the main objectives of this study are to: (i) identify spatial yield variability within the high-yielding maize dataset; (ii) understand the impacts of planting date on yield variability; (iii) explore the effect of management practices on maize yield-planting date relationship; and (iv) utilize the yield-planting date dataset collected via farmer contest-winner as a benchmarking data to be compared against a synthesis-analysis of the compendium of scientific literature available for yield-planting date relationship for the primary US maize producing regions.

## Materials and Methods

**FIGURE 1:**
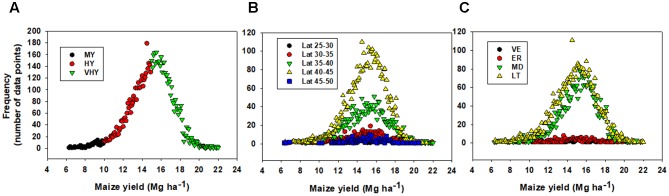
Frequency distribution of maize yield data by **(A)** yield environment, **(B)** by latitude, and **(C)** by planting date within the latitude. Medium- (5–10 Mg ha^-1^, MY), high- (10–15 Mg ha^-1^, HY), and very high-yielding (>15 Mg ha^-1^, VHY) environments. Classification as very-early (VE), early (ER), medium date (MD), and late planting date (LT) was conducted for each latitude starting with the very early planting date and grouping those planting dates within 10 days as one class, i.e., VE = first planting date in each latitude plus planting dates 10 days after the initial date.

Database for the annual farmer-driven maize contest-winner from the NCGA was collected from 2011 to 2016 period. State, county, planting date, row spacing, seeding rate, fertilizer nutrient (N, nitrogen; P, phosphorous; K, potassium) application rate, tillage, and final yield comprised the database. At the initial stage of the analysis a model, using SAS VARCOMP procedure, was fitted to quantify the variation accounted by each individual factor tested for maize yield. The model fitted in the VARCOMP procedure equates yield against random factors (year, state, irrigation, planting date, seeding rate, row spacing, tillage practice, and nutrients; **Table 1**). After determining the main factor that accounted for a relatively higher proportion of the yield variation, the data was grouped into: (1) yielding environments, i.e., 5–10, 10–15, and 15–20 Mg ha^-1^ as medium-yielding (MY), high-yielding (HY), and very high-yielding (VHY) environments, (2) latitude groups (25–30°N, 30–35°N, 35–40°N, 40–45°N, and 45–50°N) based on the county median latitude, and (3) planting date groups within each latitude [very-early (42–88 days of the year, DOY), VE; early (89–106 DOY), ER; medium (107–118 DOY), MD; and late (119–128 DOY), LT, date]. Yield frequency distribution of the data, by yield environment, latitude and planting date groups, was statistically described using SAS SUMMERY and FREQUENCY procedures (**Figure 1**).

**Table 1:** Analysis of individual effects of environment (year and state) and management factors (e.g., irrigation, planting date, seeding rate, row spacing, tillage practice, fertilization) on variance as analyzed in PROC VARCOMP procedure of SAS.

No.	Source of variation	Variance	% Variance
1	Year	0.1816	3
2	State	0.1706	3
3	Irrigation	0.0010	0
4	Planting date	0.0403	1
5	Seeding rate	0.0010	0
6	Row spacing	0.0097	0
7	Tillage	0.0010	0
8	Nitrogen	0.0022	0
9	Phosphorus	0.0031	0
10	Potash	0.0000	0
11	Error and interaction	4.9841	92
12	Total Variance	5.3945263	100


**FIGURE 2:**
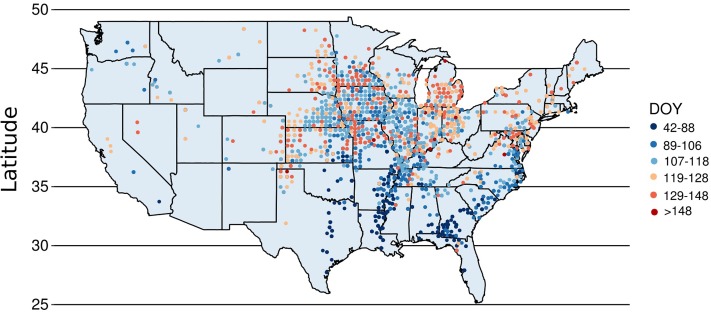
Map of US with point data portraying the specific geo-location where maize yield data was collected and individually classified as a planting date of the year (DOY) group (six groups from 42 to 88, 89 to 106, 107 to 118, 119 to 128, 129 to 148, and >148 DOY) across different latitude groups.

**FIGURE 3:**
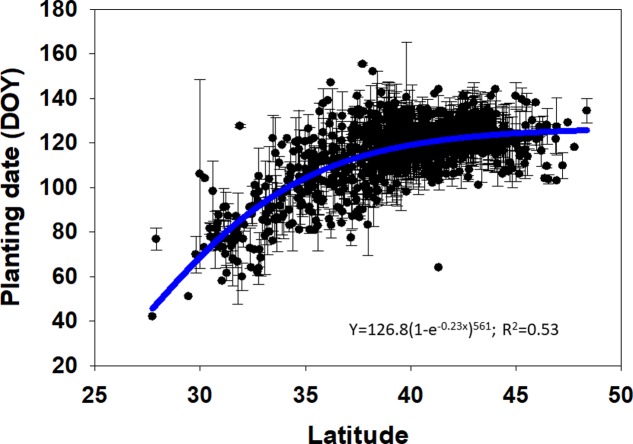
Planting date, expressed as days of the year (DOY), versus latitude for maize production within the US Midwest region, error bars represent standard error.

**FIGURE 4:**
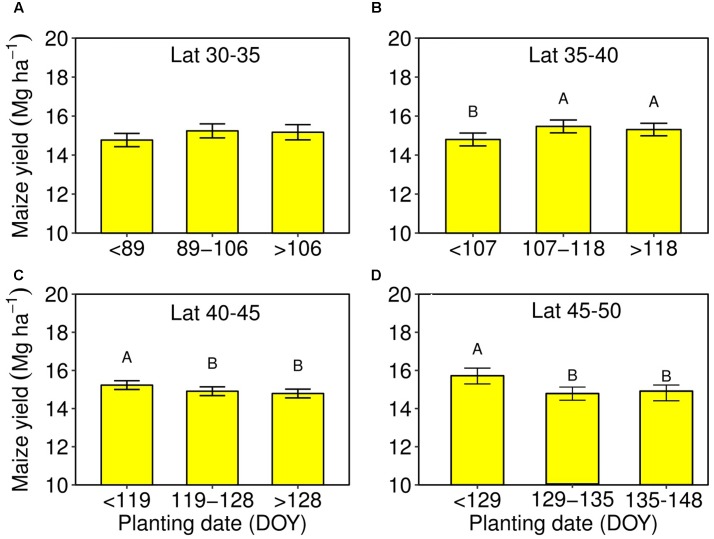
Maize yield and planting date by latitude, for the optimal planting range obtained for each latitude group from **Figure 2**, and for the range before- and after- the optimal planting window. **(A)** 30–35°N, **(B)** 35–40°N, **(C)** 40–45°N, and 45–50°N, **(D)** Error bars represent the standard error for each optimal planting window within each latitude group evaluated. Letters represent the means separation from the Tukey test comparing the planting ranges.

**FIGURE 5:**
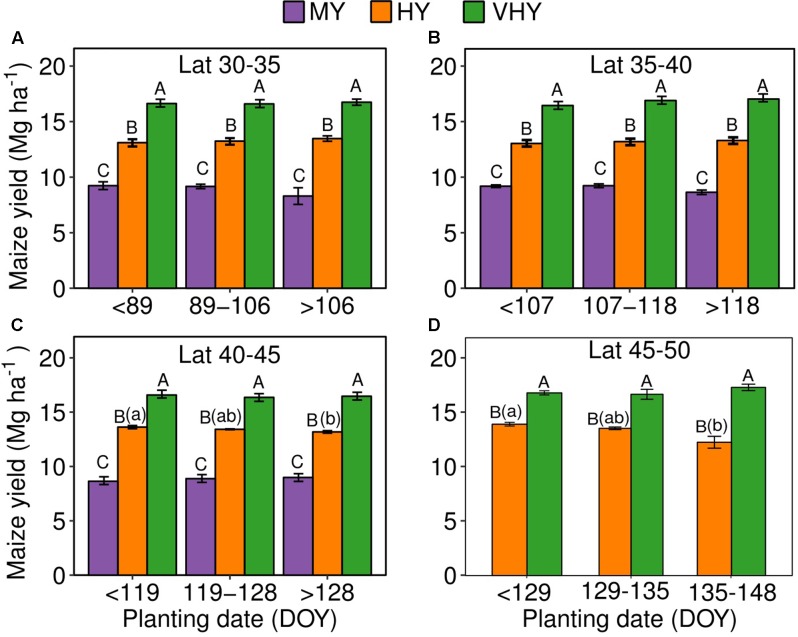
Maize yield relationship to planting date (expressed as days of the year, DOY), for the main yield environments [medium (5–10 Mg ha^-1^, MY), high (10–15 Mg ha^-1^, HY), and very-high (>15 Mg ha^-1^, VHY) yielding environments] for the latitude groups 30–35°N **(A)**, 35–40°N **(B)**, 40–45°N **(C)**, and 45–50°N **(D)**. Note that yield observations for the MY environment are not available for the 45–50°N latitude. Error bars represent the standard error for each optimal planting window within each latitude group evaluated. Letters represent the means separation from the Tukey test. Upper case letters represent comparison among the yield environments lower case letters represent comparison among planting dates.

**FIGURE 6:**
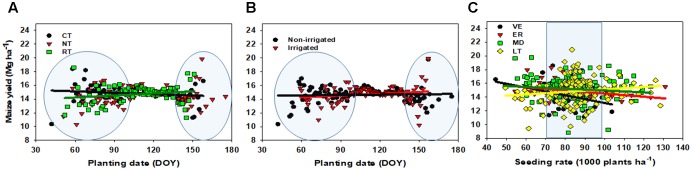
Maize yield relationship to planting date (expressed in days of the year, DOY) for conventional tillage (CT), no-tillage (NT), reduced tillage (RT) **(A)**, by irrigation, non-irrigated and irrigated **(B)**, and by seeding rate, ranging from 40 to 130 thousand per ha^-1^, for very-early (VE), early (ER), medium date (MD), and late (LT) planting dates **(C)**.

A spatial trend of the planting date was represented using “ggplot2” and “mapdata” packages in R software (**Figure 2**) (Wickham, 2009; Kahle and Wickham, 2013). The average planting date was determined for each county, and the cartographic analysis was conducted for planting date by latitude. Also, a non-linear sigmoidal model was fitted for the planting date-latitude relationship (**Figure 3**). Range of optimal planting date was further classified for each latitude based on the spatial analysis conducted and described above. Range of most frequent planting date obtained for each latitude based on the geospatial distribution of planting date (**Figure 2**) was used as base to compare planting date effect on yield. A comparison between the most frequent planting date at each latitude and earlier (before-) or later (after-) than the most frequent planting dates were compared using in SAS PROC MIXED procedure. Mean separation test, for latitudes that showed significant differences in yield by planting date at *P* = 0.05, were conducted using Tukey’s Honest Significant Difference (HSD) test. The planting date effect within each latitude was dissected by comparing average maize yield for the most frequent, earlier or later planting dates (**Figure 4**) and by yield environments (**Figure 5**) to identify if the yield trends were constant at varying yield levels. A regression analysis was conducted over the mean yields for each planting date by tillage, irrigation, and seeding rate to identify interaction of these factors (**Figure 6**).

As a last step, a synthesis-analysis of data on maize yield and planting date was collected from the scientific literature. Search criteria were established focusing on obtaining yield, planting date, geographical location and seeding rate or plant density (if available). The search engines utilized were CABI, Web of Science Core Collection, Scopus, Springer Link, Agricola and Google Scholar. Papers were retrieved using the keywords: “maize”, “planting date”, “yield”, and “US”. The criteria for inclusion of a publication were: (1) yield information available; (2) planting date; (3) geographical location; (4) year of study; –if available (5) seeding rate and/or plant density. The majority of the data were retrieved from tables and a small part from digitized figures using WebPlotDigitalizer (Rohatgi, 2012). The traits included in the database were: author, year of study, yield, planting date, geographical location (Supplementary Table S1). Units were consolidated to Mg ha^-1^ for yield, adjusted to 155 g kg^-1^ grain moisture content. For both literature and yield contest-winner data, boundary functions (0.99 quantile) (Cade et al., 1999; Ciampitti and Vyn, 2012; Koenker, 2017) were fitted in order to identify the maximum yield values attainable for each planting date. Additionally, both literature and yield contest-winner data were combined to obtain a new dataset with an intermediate yield potential. Since the yield contest-winner data (*n* = 16171) was larger than the literature data (*n* = 819), a bootstrap with replacement procedure was applied to the larger dataset to obtain two databases having equal number of observations. The same statistical procedure used in the previous two datasets was applied to the combined dataset. The statistical parameters, plateau, breakpoint and slope, from the boundary function were compared through the 95% confidence interval to check any statistically difference among them.

## Results

### Overall Variation and Yield Distribution

For maize yield, 92% of the variance was accounted for the interaction of the different factors and the error term. Environment, year, and state factors accounted for 6% of the variation in yield. The individual effect of other factors such as tillage, fertilizer, and seeding rate was zero except for planting date. The individual effect of planting date explained 1% of the variance in the yield factor from the yield contest-winner database (**Table 1**).

As for yield distribution, overall data was normally distributed with a mean of 14.8 Mg ha^-1^ and a median of 15.0 Mg ha^-1^. The distribution of the data by latitude reveals similar normality: with means of 14.7, 14.9, 14.7, 14.8, and 14.8 Mg ha^-1^ for 25–30, 30–35, 35–40, 40–45, and 45–50°N latitude groups, respectively; but with higher frequency of data for the 35–45°N relative to other latitudes groups. The distribution of the data by planting date groups portrayed means of 14.2, 15.0, 15.1, and 14.9 Mg ha^-1^ for VE (42–88 days of the year, DOY), ER (89–106 DOY), MD (107–118 DOY), and LT (119–128 DOY), respectively; but with higher data frequency for MD and LT groups (**Figure 1**). The descriptive statistical analysis allowed to confirm that, when separated in different clusters or groups based on the factors evaluated (e.g., yield environment, latitude and planting date groups), the data presented similar normality and all levels of each factor were represented in the data collected.

### US Planting Date Trend

Planting date of high-yielding US maize fields had a significant positive correlation with latitude (**Figures 2, 3**). Our analysis indicates that the most frequent range of planting date for the latitude groups between 25 and 30, 30 and 35, 35 and 40, 40 and 45, and 45 and 50°N was 42 and 88, 89 and 106, 107 and 118, 119 and 128, and 129 and 135 DOY, respectively for each latitude range evaluated (**Figure 2**). These ranges of most frequent planting dates suggest that planting date in lower latitudes is not only earlier in the year but also has a wider window, without losing yield, compared to the higher latitudes (e.g., 46 days for 25–30°N vs. 6 days for 45–50°N). Model fitting for the relationship between planting date and latitude, in a continuous manner (not in latitude ranges), followed a sigmoidal curve (*R*^2^= 0.53; **Figure 3**). A sharp change in planting dates from 60 to 100 DOY was documented as latitude increases from mid-20 to 35°N, but presenting a small change from 100 to 120 DOY as latitude increases from 35 to 50°N. In agreement, the sigmoidal curve fitted for planting date and latitude portrayed a wider planting date between 35 and 40°N compared to higher latitudes.

### Planting Date Effect on Yield by Latitude and by Yield Environment

The importance of planting date on yield was apparent in high latitudes (above 40°N) compared to lower latitudes (below 40°N). In the lower latitude range, 30–35°N, there was no significant difference for maize yield between the most frequent range of planting date, 89–106 DOY, and less than or above the most frequent planting time (**Figure 4A**). In the mid latitude range, 35–40°N, yield was reduced when planting was anticipated relative to the most frequent range of planting date, 107–118 DOY (**Figure 4B**). Comparisons of yield at the most frequent planting date, below or above the range of planting dates reflects a yield penalty when planting was delayed after 119 DOY for 40–45°N and after 129 DOY for 45–50°N (**Figures 4C,D**).

For the effect of the yield environment factor, as expected, a similar pattern was encountered at all latitude groups with MY < HY < VHY (**Figure 5**). In lower latitude groups (30–35 and 35–40°N), no obvious yield difference was observed between planting dates of the HY and VHY environments (**Figures 5A,B**). In the 40–45°N latitude, a more variable effect of planting date was documented for the HY group, slightly declining (0.4 Mg ha^-1^) from the early to the late (and less frequent) planting date ranges (**Figure 5C**). In the 45–50^o^N latitude, a similar yield decline (1.3 Mg ha^-1^), relative to the 40–45°N group, was observed for the HY environment with a similar reduction in yield when planting moved from the earliest to the latest planting date window (**Figure 5D**).

### Planting Date Interaction with Other Management Factors

The effect of tillage, irrigation, and seeding rate on yield and planting date relationship was pursued by a descriptive analysis (**Figure 6**), due to their lower proportion of variance accounted for the yield factor (**Table 1**). For the tillage factor, if planting is early (<90 DOY), conventional tillage (CT) seemed to outyield reduced tillage (RT); without presenting a clear yield difference right after this planting date (>90 DOY). As planting is delayed (>130 DOY), no-till (NT), or RT portrayed a trend to outyield CT (**Figure 6A**). The latter could be partially explained by water supply and drainage factors. When planting is delayed, RT operations might contribute to conserving water and, in consequence, improve yields. For the irrigation factor, a trend of superior yield with early planted non-irrigated was recorded relative to the irrigated environment. When planted late, the above-mentioned yield trend reverses itself (**Figure 6B**). Lastly, for the yield to the seeding rate relationship, each planting date group resulted in: (i) VE, negative association for yield as seeding rate increases, (ii) ER, slightly negative, (iii) MD, almost not responsive, and (iv) LT, slightly positive (**Figure 6C**).

### Synthesis-Analysis Planting Date and Yield Relationship

**FIGURE 7:**
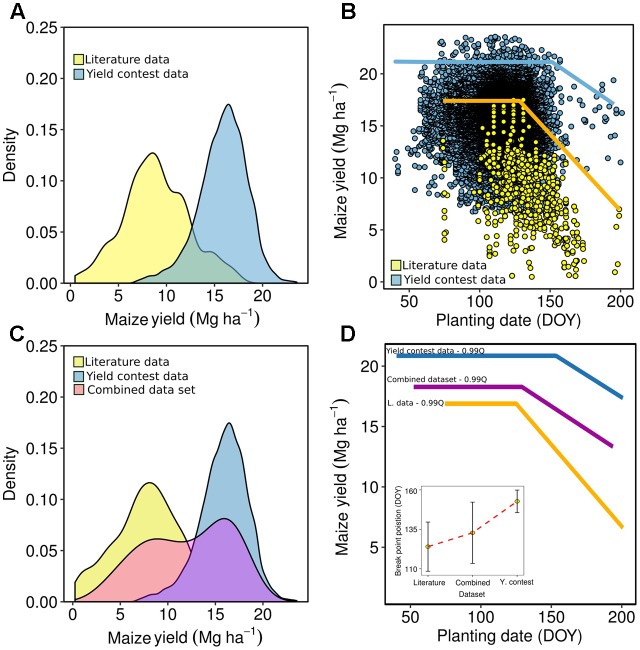
Yield frequency of the literature (Supplementary Table S1) and yield contest data **(A)**, frontier analysis (bi-linear models, 99% quantile) for the yield and planting date effect for literature and the dataset from the maize contest-winners from National Corn Growers Association (NCGA) **(B)**, yield frequencies considering literature, yield contest, and combine data sets **(C)**, and frontier analysis for all three data sets: literature, yield contest, and combined **(D)**. Inset for panel **(D)** refers to the break point of the linear-plateau model for the literature, combined, and yield contest datasets, with error bars depicting the 95% confidence interval.

The yield-planting date dataset collected via farmer-driven contest-winner was utilized as a benchmarking data to be compared against the compendium of scientific literature available for yield and planting date relationship for the primary US maize producing regions (Supplementary Table S1). The overall yield for the literature data presented a lower mean relative to the yield contest-winner data (**Figure 7A**) with a more widespread yield distribution from low to high yield values. A frontier analysis (99% quantile linear-plateau model) was fitted to each dataset (**Figure 7B**). Both the literature and yield contest-winner datasets fitted a bi-linear model but presenting different even breakpoints for planting date (123 and 152 DOY, respectively), maximum yield values (yield plateau at 18 and 21 Mg ha^-1^, respectively), but not statistical difference was documented for yield reduction per unit of DOY after the even breakpoints. Thus, as the maximum attainable yield increases the effect of planting date as a main critical factor becomes less relevant. The latter was reflected as a longer plateau (planting date window for maximum yield), a superior even breakpoint (DOY), and with similar (relative to the literature data set) reduction per unit of DOY as planting date was delayed. Both the literature and yield contest-winner datasets were aggregated to form a unified dataset, herein termed as combined model (**Figure 7C**). Frontier functions for all three datasets (contest-winner, combined, and literature) reflected a transition of the planting date effect on maize yields, from a greater impact when maximum attainable yield was lower (literature data) to a lower effect as yield potential increases (contest-winner data), but not statistical differences were documented for breakpoints and slopes for both literature and contest-winner data. (**Figure 7D**).

## Discussion

Identification of optimum planting date or planting window has been one of the most important practices by farmers to have a good crop establishment and harvest. Previous studies on planting date association with yield variation for the last three decades (1979–2014) in US were summarized in Supplementary Table S1 showed that for northern mid-latitudes (Illinois) planting date occurs from April to May, 1997 and 2006. Focused on 12 central Corn Belt states, Kucharik (2006) found an overall trend for a 2-week earlier initiation of maize planting date in 2005 compared to early 1980s. A spatial distribution for US maize planting date was synthesized in **Figure 2**. Planting date was positively correlated with latitude and most frequent planting window differed by latitude group (**Figure 3**). No significant impact was found for wider planting date on yield at the lower latitude group (30–35°N) (**Figure 4**). However, majority of high-yielding maize fields were planted between 89 and 106 DOY (between March and April). The latter is in agreement with Wiatrak and Wright (2004) in a study at Quincy, FL (31°N), with the highest yield of 9.6 Mg ha^-1^ obtained when maize was planted from March to April. Bruns and Abbas (2006) supported that best planting date to obtain highest yield (9.2 Mg ha^-1^) for maize at Stonville, MS (33°N) was around mid-April.

Favorable planting window was clearly shifted to later dates for the mid- (35–40°N) compared to the lowest latitude (30–35°N) group. In the mid-latitude group, farmers still had a wider planting window from 107 to 118 DOY (mid-April to end of April) for high-yielding maize production. Our result supports previous findings from similar latitudes that planting date for high-yielding maize could be obtained when planted from April to mid-May (Supplementary Table S1). Norwood (2001) showed that early May planting produced greater yield than mid-April under a dryland condition in Garden City, KS (38°N), United States. However, in this latitude group the planting date window should not be later than mid-May. For a similar latitude, delaying maize planting time to 128–135 DOY resulted in a yield reduction probably due to a shorter growing season or greater incidence of a frost before maturity for full-season maize hybrids (Grassini et al., 2011).

Extensive studies have been conducted to identify the best planting date or favorable planting window at the higher latitude groups (40–45°N) (Supplementary Table S1; Kucharik, 2006, 2008; Sacks et al., 2010). Significant yield reduction was found when maize planting time was delayed after 119 DOY (beginning of May) for 40–45°N and after 129 DOY (mid-May) for 45–50°N (**Figures 4, 5**). Studies conducted in similar latitudes (Supplementary Table S1) showed variation in yield-planting date responses within states. However, the variation in planting window found within states was not substantial and favorable planting window for high-yielding maize systems was found in the literature similar to our finding, occurring from late-April to mid-May. Early planting date (before mid-April) will be most likely to be affected by freezing soils, cooler soil temperatures for adequate plant emergence and early establishment (Bollero et al., 1996; Swanson and Wilhelm, 1996; Kucharik, 2006, 2008), and wet early season soil conditions.

Favorable planting window documented for our study for different yield environments (**Figure 5**) is in agreement with previous literature published in the last three decades, especially for the high latitude (from 40°N) (Supplementary Table S1), suggesting that the data submitted by farmers in yield contests and methods presented here are valuable for providing guidance on identifying optimal planting windows for high-yielding maize production. As farmers participating in the yield contests were considered to implement best production practices, the factors that are associated with the data could provide guidance for advancing crop management (Cassman, 2016).

Management factors including genotype (crop maturity, drought and conventional, Bt and non-Bt), tillage, residue rate, plant density, fertilizer N, weed control, pest and diseases were studied in the scientific literature related to planting date since 1979 to 2014 (Supplementary Table S1). Our findings are in agreement with Grassini et al. (2011) with that planting date, tillage system and plant density (a trait associated to seeding rate) were the most sensitive factors affecting yield. Tillage was a relevant factor when planting for maize was outside of the optimal planting window. For the northern US, CT tillage provided good soil conditions (warmer); therefore, superior maize yield was found as comparing to NT (colder soil) when planting early (119 DOY) (Imholte and Carter, 1987). When planting was delayed, RT might contribute to conserving water and relatively improving yield (**Figures 6B,C**). Thus, water availability seems to be a main determinant for choice of planting date and tillage systems. When planted early, soil temperature and moisture conditions are the main factors affecting maize stand establishment and yields regardless of the tillage system (Perez-Bidegain et al., 2007). At high latitude, the highest yields were obtained when planting in early May under both tillage systems (CT and NT) at Arlington, Wisconsin (Imholte and Carter, 1987). As for the seeding rate factor, early and late planting date groups were associated to lower and higher seeding rates (**Figure 6C**), but could be primarily explained by two reasons. The first one is the possibility of yield compensation from additional ear, number of grains per ear, and grain weight when planted early relative to a late planting date. The second one is when planting was completed early, long maturing hybrids require less plant density for similar or better yield than the short maturing hybrids. Our results are in agreement with Lindsey and Thomison (2016) that reported an increase in the agronomic plant density to produce maximum yield when planting was delayed. In addition, Norwood (2001) found in Kansas that late planting date (early May) combined with superior plant density provided greater yield than late April with lower plant density. In contrast, but in agreement with our finding in the optimum planting window (108–117 DOY), no interaction between planting date and seeding rate/plant density was documented in Illinois (Nafziger, 1994) and in southern Minnesota (van Roekel and Coulter, 2011).

Our synthesis-analysis shows yield to planting date relationship fitted a bi-linear model for both yield contest-winner and the literature datasets but with shorter duration on the optimal planting window to maximize yields as the maximum yield was reduced. For the synthesis-analysis, the literature (Supplementary Table S1) and contest-winner datasets presented a similar bi-linear model but with different even breakpoints and maximum yield values (plateau-levels). Thus, as the maximum attainable yield increases then the effect of planting date as a main critical factor become less relevant. A combined model was produced between the literature and yield contest winner data, herein termed as combined model, presenting a yield distribution in between both datasets (**Figure 7**). The latter might reflect that on those high-yielding environments, planting date is not the primary limiting factor; but it becomes more important as the latitude increases and when planting time was delayed relative to the most frequent range of planting date determined within each latitude group.

## Conclusion

Maize yield farmer-driven contest-winner data provided the opportunity to produce a comprehensive planting date map for high-yielding maize across different US latitude groups. Key outcomes of this synthesis-analysis were: (i) a tighter planting window was found at higher latitude (above 40°N, end-April to mid-May) than at lower latitude groups; (ii) maize yield was maximized when the planting window was 89–106 DOY for the 30–35°N, 107–118 DOY for the 35–40°N, <119 DOY for 40–45°N, and <129 DOY for 45–50°N, and (iii) in overall, yield to planting date relationship fitted a bi-linear model for both the contest-winner and the literature data but with shorter planting window duration to maximize yields as the yield was reduced.

For this synthesis-analysis, both the contest-winner and literature datasets portrayed that planting date becomes a more relevant factor for high-yielding US maize systems when planting late with a shorter planting window duration in high- relative to low-latitudes.

## Author Contributions

Contributed to conception or design of the work: IC, YA, and NL. Collected the data: NL. Contributed in data analysis and interpretation: YA, NL, RS, and IC. Drafted the article: NL, YA, and RS. Contributed in critical revision of the article: IC. Final approval of the version to be published: IC.

## Conflict of Interest Statement

The authors declare that the research was conducted in the absence of any commercial or financial relationships that could be construed as a potential conflict of interest.

## Abbreviations

DOY: days of year
Planting date groups: VE, ER, MD, LT are very early, early, medium, and late
Tillage systems: CT, NT, and RT are Convention till, No-till, and Reduced tillage
Yield environments: MY, HY, VHY are medium-, high-, and very-high
