# Supplementary material for: Maize Yield and Planting Date Relationship: A Synthesis-Analysis for US High-Yielding Contest-Winner and Field Research Data
Source: Front Plant Sci. 2017 Dec 21;8:2106. doi: 10.3389/fpls.2017.02106 (PMC5743010; doi:10.3389/fpls.2017.02106)
Supplement: Supplementary file 1 [file Table_1.DOCX]

**Maize Yield and Planting Date relationship: A synthesis-analysis for US High-Yielding Contest Winner and Field Research Data**

Supplementary Material

Nguyen Viet Long^12^, Yared Assefa^1^, Rai Schwalbert^1^, and Ignacio A. Ciampitti^1*^

*** Correspondence:** [Ciampitti@ksu.edu](mailto:Ciampitti@ksu.edu)

**Table S1.** Maize yield- planting date relationship studies in USA from 1979 to 2014 period listed by low to high latitudes. Search criteria were established focusing on obtaining yield, planting date, geographical location and a measurement of plant density or seeding rate (if available). The search engines utilized were CABI, Web of Science Core Collection, Scopus, Springer Link, Agricola and Google Scholar. Papers were retrieved using the keywords: “maize”, “planting date”, “yield”, and “US”. The majority of the data was retrieved from tables, some from equations, and apart from digitized figures using WebPlotDigitalizer (Rohatgi, 2012). The following information were included author/year of publication, location/state (US), latitude, longitude year of research, yield range, planting date range, and factors evaluated in each study. A study is relisted if having more than one location.

| **Author/Year of publication** | **Location/**  **State (US)** | **Latitude** | **Longitude** | **Year of research** | **Yield range (Mg^-1^ ha)** | **Planting date range (DOY)** | **Factors evaluated** |
| --- | --- | --- | --- | --- | --- | --- | --- |
| Wiatrak and Write, 2004 | Quincy, FL | 31 | -85 | 1998-2001 | 0.2-9.6 | 74-228 | Hybrid types e.g Bt and none Bt, late planting date in US Southeast |
| Bruns and Abbas, 2006 | Stonville, MS | 33 | -91 | 2004 | 7.8-9.1 | 96-131 | 12 hybrids Bt and non-Bt corn with differences in maturity |
| Norwood 2001 | Garden city, KS | 38 | -101 | 1996-1999 | 2.5-9.9 | 105-132 | Hybrids, plant population, drought tolerant maize |
| Bollero and Hollinger,, 1996 | Urbana, IL | 40 | -88.2 | 1992-1993 | 6.6-9.1 | 128-158 | Controlled low soil temperature under field conditions |
| Anapalli et al., 2005 | Akron, CO | 40 | -103 | 1991-1993 | 3.3-9.7 | 116-170 | Short, mid- and long-season hybrids simulation study |
| Grassini et al., 2011 | Holdrege, NE | 40 | -99 | 2001-2010 | 14.3-17.5 | 110-131 | Yield potential and agronomic practices, simulation study |
| Gower et al., 2002 | Wooster, OH | 41 | -81.9 | 1998-1999 | 6.1-12.8 | 124-155 | Weed control and glyphosate postemergence application |
| Lindsey and Thomison, 2016 | Wooster, OH | 41 | -81.9 | 2012-2014 | 111.08-14.14 | 122-153 | Drought tolerant vs conventional hybrids, plant population |
| Swanson and Wihelm, 1996 | Lincoln, NE | 41 | -96.7 | 1986 | 3.3-6.9 | 122-160 | Residue rate |
| Nafziger 1994 | Monmouth, IL | 41 | 90.6 | 1987-1990 | 5.7-10.9 | 100-144 | Plant population study |
| Gower et al. 2002 | Charleston, OH | 41 | -81 | 1998-1999 | 7.1-13.2 | 114-146 | Weed control and glyphosate postemergence application |
| Lindsey and Thomison 2016 | Charleston, OH | 41 | -81.2 | 2012-2014 | 11.8-14.9 | 135-153 | Drought tolerant vs conventional hybrids, plant population |
| Lindsey and Thomison 2016 | Hoytville, OH | 41 | -83.8 | 2012-2014 | 11.3-11.6 | 130-161 | Drought tolerant vs conventional hybrids, plant population |
| Abendroth et al., 2017 | Crawfordville, IA | 41 | -91.5 | 2006-2009 | 9.3-12.1 | 103-152 | Planting window for Iowa |
| Russelle et al., 1987 | Mead, NE | 41 | -96.5 | 1979 | 7.8-10.7 | 115-150 | Nitrogen use efficiency |
| Abendroth et al., 2017 | Lewis, IA | 41 | -95.1 | 2006-2009 | 13.0-9.5 | 100-152 | Planting window for Iowa |
| Perez-Bidegain et al., 2007 | Newton, IA | 42 | -93 | 2002-2004 | 9.4-14.8 | 101-139 | Tillage system |
| Nafziger, 1994 | Dekalb, IL | 42 | -88.8 | 1987-1990 | 5.8-10.6 | 100-144 | Plant population |
| Lauer et al., 1999 | Lancaster, WI | 43 | -91 | 1991-1994 | 5.0-14.0 | 109-162 | Full and short season hybrids |
| Abendroth et al., 2017 | Kanawha, IA | 43 | -93.8 | 2006-2009 | 7.23-11.43 | 106-153 | Planting window for Iowa |
| Abendroth et al., 2017 | Nashua, IA | 43 | -92.5 | 2006-2009 | 10.5-13.2 | 101-153 | Planting window for Iowa |
| Abendroth et al. 2017 | Sutherland, IA | 43 | -95.5 | 2006-2009 | 9.7-11.1 | 106-153 | Planting window for Iowa |
| Imholte and Carter 1987 | Arlington, WI | 43 | -89.4 | 1983-1985 | 5.1-9.2 | 125-154 | Tillage system |
| Lauer et al. 1999 | Arlington, WI | 43 | -89 | 1991-1994 | 1.8-13.8 | 110-175 | Full and short season hybrids |
| Van Roekel and Coulter, 2011 | Lamberton and Waseca, MN | 44 | -93.50 | 2008-2010 | 10.6-12.6 | 120-150 | Plant density, agronomic practices |
| Lauer et al., 1999 | Hancock,  WI | 44 | -90 | 1991-1994 | 3.8-12.6 | 108-163 | Full and short season hybrids |
| Lauer et al., 1999 | Marshfield, WI | 45 | -90 | 1991-1994 | 0.3-11.2 | 112-164 | Full and short season hybrids |
| Lauer et al., 1999 | Spooner, WI | 46 | -91.89 | 1991-1994 | 0.9-8.2 | 111-169 | Full and short season hybrids |
| Lauer et al., 1999 | Ashland, WI | 47 | -90.9 | 1991-1994 | 1.1-12.5 | 116-163 | Full and short season hybrids |
